# Characterization of Flavonoid Compounds in Common Swedish Berry Species

**DOI:** 10.3390/foods9030358

**Published:** 2020-03-19

**Authors:** Jiyun Liu, Mohammed E. Hefni, Cornelia M. Witthöft

**Affiliations:** 1 Department of Chemistry and Biomedical Sciences, Faculty of Health and Life Sciences, Linnaeus University, 392 31 Kalmar, Sweden; cornelia.witthoft@lnu.se (C.M.W.); mohammed.hefni@lnu.se (M.E.H.); 2 Food Industries Department, Faculty of Agriculture, Mansoura University, P.O. Box 46, Mansoura 35516, Egypt

**Keywords:** extraction, flavonoids, HPLC-UV/MS, polyphenols, Swedish berries

## Abstract

Berries are considered an ideal source of polyphenols, especially from the flavonoid group. In this study, we examined the flavonoid content in 16 varieties of Swedish lingonberry, raspberry, blueberry, and strawberry. Nineteen flavonoids were simultaneously quantified using external standards. An additional 29 flavonoids were tentatively identified using MS as no standards were available. Quantification was done using HPLC-UV after optimization of chromatographic and extraction procedures. The method showed high linearity within the range of 2–100 μg/mL (correlation co-efficient >0.999), intra- and inter-day precision of 1.7–7.3% and average recovery above 84% for all compounds. Blueberries and lingonberries were found to contain higher contents of flavonoids (1100 mg/100 g dry weight) than raspberries and strawberries (500 mg/100 g dry weight). Anthocyanins were the dominant flavonoids in all berries. The tentatively characterized compounds contribute 18%, 29%, 61%, and 67% of the total flavonoid content in strawberries, lingonberries, raspberries, and blueberries, respectively. Overall, Swedish berries were shown to be good sources of polyphenols.

## 1. Introduction

Berries are recognized as a good source of flavonoids. Flavonoids, a primary subgroup of polyphenolic compounds, have been shown to possess potent antioxidant, antimicrobial, and anti-inflammatory properties [1,2,3] and to exhibit beneficial effects against obesity, diabetes, neurodegenerative disorders, and cardiovascular disease [4,5,6,7]. Four flavonoid groups, anthocyanins, flavonols, flavan-3-ols, and proanthocyanidins, commonly exist as plant secondary metabolites in nature. The anthocyanins are pigment compounds in glycosylated forms that affect the colors present in growth periods [8]; the flavonols exist widely in fruits and berries in glycosylated or acetylated forms; the flavan-3-ols include (+)-catechin, (−)-epicatechin, gallocatechin, and epigallocatechin as dominant monomers; and the proanthocyanidins are polymers of A- and B-type, based on the location of interflavan linkages [9]. The structural diversity among flavonoids gives rise to their different chemical characteristics, physiological benefits, and pharmacokinetic behaviors.

Besides flavonoids, berries have also been reported to contain phenolic acids, which occur dominantly in the bound form. However, in some studies, both, flavonoid compounds and phenolic acids were simultaneously extracted using the same extraction method [10,11,12,13]. Complete extraction of flavonoids and free phenolic acids can be achieved using organic solvents [14,15], whereas acid or alkaline hydrolysis at high temperature is normally used for extraction of bound and insoluble phenolic acids [16,17].

With respect to flavonoid quantification, several methods for extraction have been developed. Ultrasonic-assisted extraction methods are recommended owing to the high reproducibility during analysis and low cost in terms of both time and energy [18]. Extraction conditions, which include the type of solvents used for extraction, the ratio of solvent to sample, the number of extraction repetitions, and the duration of extraction, are also factors that affect eventual outcomes [16,19]. For example, Pereira et al. [20] and Kylli et al. [21] even used two-step extraction procedures during quantification of groups of flavonoids in berries. High performance liquid chromatography (HPLC) is a favored and widely applied technique for quantification, and acidified water and acetonitrile are often chosen as the mobile phase [16].

A wide range of flavonoid content (105–1730 mg/100 g fresh weight) in berries has been reported, depending on the type, variety, and growing conditions [22,23,24]. However, information regarding flavonoid composition of some specific berries commonly grown in Sweden is limited (e.g., lingonberry) or lacking (e.g., strawberry variety “Favori”).

The aims of the present study were to (1) optimize a procedure for extraction of flavonoids in berries for analysis using HPLC-UV/MS, and (2) to quantify the flavonoid content in several varieties of Swedish lingonberry, raspberry, blueberry, and strawberry.

## 2. Materials and Methods

### 2.1. Reagents, Standards, and Solvents

Nineteen flavonoid compounds (Table 1) were purchased from Extrasynthese (Genay, France). Methanol (HPLC grade, ≥99.9%) was purchased from Honeywell (Seelze, Germany), HPLC-grade acetonitrile from VWR international (Stockholm, Sweden), ethanol (AR, 99.5%) from Solveco (Rosersberg, Sweden), formic acid (ACS, 98–100%) from Merck KGaA (Darmstadt, Germany), and acetone from Sigma-Aldrich (St. Louis, MO, USA).

Stock standard solutions were prepared by dissolving the individual flavonoid compounds in methanol to reach a final concentration of 1000 μg/mL. All solutions were kept under nitrogen protection and stored in darkness at 4 °C.

### 2.2. Berry Samples

Sixteen berry samples commercially available in the Kalmar area, southern Sweden, were purchased in summer 2018. Information regarding variety (except lingonberry) was received from the producers. These comprised wild lingonberries (*Vaccinium vitis-idaea*) from two producers (unknown varieties, here named L1 and L2), strawberries (*Fragaria ananassa*) of seven varieties (namely Evie, Faith, Favori, Malwina, Rumba, Salsa, and Sonata), blueberries (*Vaccinium myrtillus*) of four varieties (Bluecrop, Camelia, Duke, and Legacy), and raspberries (*Rubus idaeus*) of three varieties (Glen Ample, Kweli, and Versalle). 

Based on the popularity of lingonberry in European countries and the diversity of its flavonoid profile [25], one of the lingonberry samples (L1) was selected as the in-house control sample to optimize the extraction method.

All berry samples were separately kept in polyethylene bags and stored at −20 °C before lyophilization. After freeze drying (BenchTop Pro, VirTis, USA), the samples were milled using a laboratory-scale mill (Cyclotec 1093, Tecator, Sweden) and stored at −20 °C until further analysis within a week.

### 2.3. Sample Extraction

Extraction conditions (i.e., the extraction solvent, the number of extraction repetitions, and the ratio of solvent volume to sample weight) and reconstitution solvent (for dissolving dried extracts prior to injection into HPLC) were optimized based on the method of Latti et al. [26] using the in-house control sample lingonberry L1. 

The effects of several types of extraction solvents (aqueous methanol, ethanol, or acetone at a concentration of 50%, 70%, and 100% (*v*/*v*) with the addition of formic acid (0%, 1%, 3%, and 5%, *v*/*v*)) on flavonoid yield were studied with a solvent volume to a sample weight ratio of 15 μL/mg). To optimize the number of extraction repetitions, flavonoids in the in-house control sample were extracted in four repetitions and the extract from each repetition was analyzed separately. To optimize the reconstitution solvent, the different standard solutions were used. Five different methanol concentrations (100%, 80%, 50%, 40%, and 30% in water, (*v*/*v*)) were investigated for reconstitution using standards for individual compounds (10 μg/mL).

In the optimized extraction procedure 210 μL methanol was added to 14 mg freeze-dried berry (*n* = 3). Samples were sonicated for 15 min prior to centrifugation for 5 min at 13,000× rpm. The supernatants were collected, while the pellets were re-extracted another two times using the same procedure. The combined supernatants were concentrated using SpeedVac (SC100, Thermo Scientific, Waltham, MA, USA) at a medium temperature (43 °C) until dryness. The dry residue was redissolved in methanol/water (40:60, *v*/*v* and volume/mass = 30), and filtered through a 0.45 μm Millipore filter (Agilent, St. Clara, CA, USA) before analysis by HPLC-UV.

### 2.4. Quantification

Quantification of flavonoids was carried out using HPLC-UV/MS (Agilent 1200 series, St. Clara, CA, USA) with the Agilent OpenLab Software Suite Rev. C.01.07. The mass spectrometer (Agilent 6130 Quadrupole, St. Clara, CA, USA) was fitted with electrospray ionization (ESI) and operated in a positive ion mode. Parameters were set as follows: drying gas flow 11.0 L/min, nebulizer pressure 55 psig, drying gas temperature 250 °C, and capillary voltage 3000 V. Mass spectra in the range of mass-to-charge ratio (*m/z*) 285–670 were collected. 

Flavonoids were separated on a 250 mm × 4.6 mm, 3 μm, Luna^®^ Omega C18 column (Phenomenex, Torrance, CA, USA) fitted with a 4 mm × 3.0 mm, C18 Security Guard Cartridge (Phenomenex, Torrance, CA, USA). The column temperature was set to 40 °C, the injection volume to 20 μL, and the flow rate to 1 mL/min. Several mobile phase compositions were investigated: 1%, 3%, 5%, 7%, 10%, and 12% formic acid in water as solvent A; and acetonitrile/methanol/water (90:5:5, 85:7.5:7.5, and 80:10:10 *v*/*v*/*v*) as Solvent B, based on the method of Vagiri et al. [27]. Finally, 3% formic acid in water was employed as solvent A and acetonitrile/methanol/water (80:10:10, *v*/*v*/*v*) as solvent B. The optimized gradient was as follows: 0–45 min, linear gradient from 5% to 29% B; 45–46 min, linear gradient from 29% to 50% B; 46-48 min, 50% B isocratic; 48–49 min, linear gradient from 50% to 5%; 49–55 min, 5% B isocratic. 

Quantification was based on an external multilevel calibration curve (*n* = 6) of 19 flavonoids at 280 nm for flavan-3-ols, 360 nm for flavonols and 520 nm for anthocyanins, according to Vagiri et al. [27]. For tentative identification of further peaks, which according to the literature were expected in the berry extract and where no standards were available, mass spectrometry (Agilent 1200 series, St. Clara, CA, USA) was used; anthocyanins were quantified using UV against cyanidin-3-*O*-glucoside (520 nm), flavonols against quercetin-3-*O*-galactoside (360 nm), B-type proanthocyanidin dimers against procyanidin B1 (280 nm), and A-type proanthocyanidin dimers against procyanidin A2 (280 nm) [15,28].

### 2.5. Quality Control 

Linearity of each calibration curve (*n* = 6) within the range 2–100 μg/mL was evaluated by linear regression analysis. Limit of detection (LOD) and limit of quantification (LOQ) of the compounds identified were determined from the calibration curve data as: LOD = (3.3 × SD)/b; LOQ = (10 × SD)/b, where SD is the residual standard deviation of the linear regression and b is the slope of the regression line [29]. 

Extraction recovery was investigated by addition of an upper (100% of the expected concentration in samples) and a lower (50% of the expected concentration in samples) level of standards to the in-house control sample prior to extraction. Recovery (*R*, %) was calculated as: *R* = 100 × (C_found_−C_sample_)/C_added_, where C_found_ indicates the content measured after addition of standard compounds, C_sample_ indicates the content measured before addition, and C_added_ indicates the added amount of standard compounds.

The intra-day variation was calculated from the triplicate assays of the lingonberry extract on the same day (*n* = 3), while the inter-day variation was measured from assays of the same batch for three separate days (*n* = 3). The results were expressed as coefficient of variation (CV, %) of means for peak area.

Stability of authentic standard compounds at two different concentrations (5 and 50 μg/mL) was evaluated after storage at 4 °C in darkness for three months. The stability was monitored twice every month for three months by comparing HPLC peaks of standards before storage and after each storage time point.

### 2.6. Calculations and Statistical Analysis

The total amount of flavonoids (mg/100 g dry weight (dwt) of freeze-dried berries) in each berry species was calculated as the sum of the four subgroups (i.e., anthocyanins, flavonols, flavan-3-ols, and proanthocyanidin dimers), including the tentatively identified compounds. All results were expressed as mean ± SD. Linearity of calibration curves was determined using regression analyses (Excel, Microsoft, Redmond, WA, USA). Flavonoid yield when optimizing extraction was compared using one-way analysis of variance (ANOVA), significance was set to *p* < 0.05 (Prism 8, GraphPad, La Jolla, CA, USA).

## 3. Results and Discussion

### 3.1. Method Optimization for Berry Matrix

**Figure 1:**
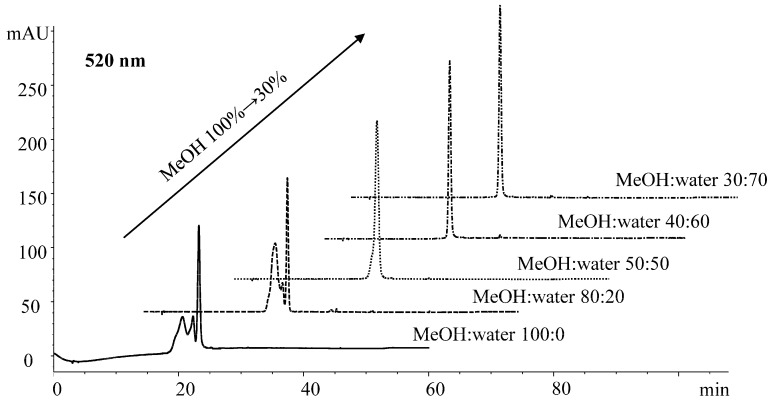
Effect of the reconstitution solvent on peak shape as exemplified by cyandin-3-*O*-glucoside standard (10 μg/mL) at 520 nm. HPLC conditions as described in Section 2.4.

With regard to optimization of the reconstitution solvent for compounds before HPLC quantification, a high percentage of methanol in the reconstitution solvent resulted in distortion of peaks (Figure 1). Using 40% and 30% methanol in water (*v*/*v*) as the reconstitution solvent achieved peaks with symmetry around 1.0 without distortion or tailing. The observed solvent effect is in line with findings by Mirali et al. [30] that a high organic proportion in the reconstitution solvent tends to have an adverse effect on chromatography. 

**Figure 2:**
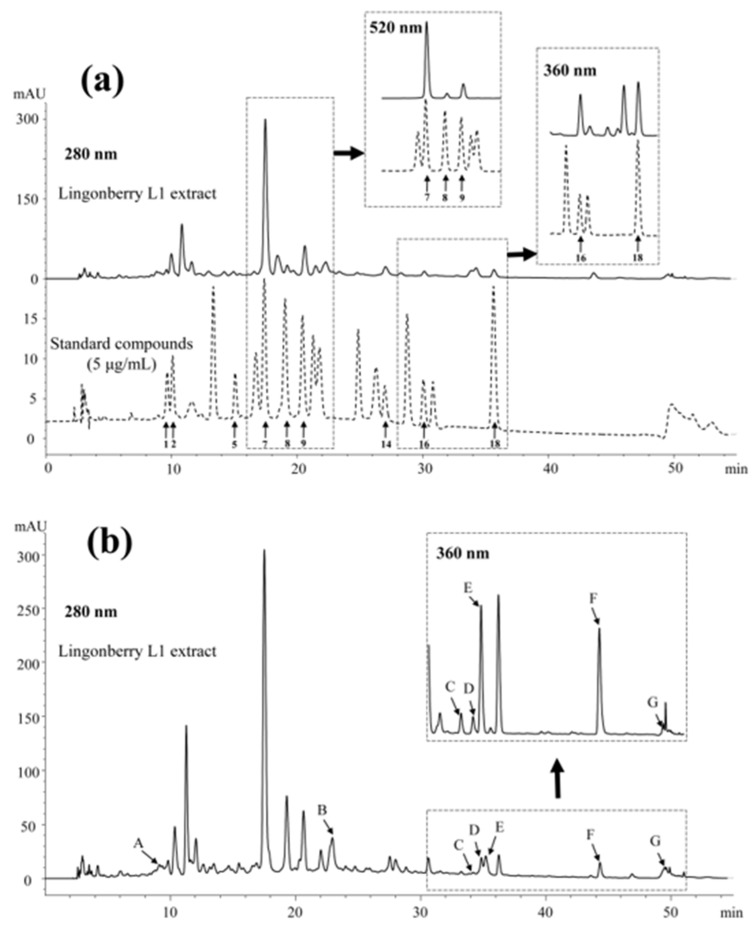
Chromatograms of lingonberry L1 extract and flavonoid standards (5 μg/mL) at 280 nm. (**a**) Flavonoids quantified using authentic standards. Peak numbers (1–18) refer to compounds listed in Table 1. (**b**) Flavonoids tentatively identified and quantified. A, B-type procyanidin; B, A-type procyanidin; C, quercetin-3-*O*-xyloside; D, quercetin-3-*O*-arabinoside; E, quercetin-3-*O*-arabino-furanoside; F, quercetin-3-*O*-(4’’-HMG)-rhamnoside; G, kaempferol-(HMG)-rhamnoside. The concentration of flavonoids in lingonberry extract ranged from 1.5 to 101.9 μg/mL.

With respect to mobile phase composition, an increase in formic acid proportion up to 12% (*v*/*v*) significantly improved peak separation and prevented tailing (Figure S1 in Supplementary Materials). Quercetin-3-*O*-rhamnoside and kaempferol-3-*O*-glucoside could not be separated unless the concentration of formic acid was above 5%, while they were completely separated when the concentration was above 7%. Thus use of a higher percentage of formic acid in the mobile phase was preferable in flavonoid analysis, which is in agreement with recommendations by Vagiri et al. [27]. However, considering the recommended working pH of the column (pH 1.5–8.5), a compromise was made and 3% formic acid in water (pH 1.92) was finally selected as solvent A. As for solvent B, use of acetonitrile/methanol/water with composition 80:10:10 (*v*/*v*/*v*) resulted in higher peak resolution (Figure 2) than with the two other solvents (data not shown), and thus it was chosen as the optimal mobile phase B. Using optimized chromatographic conditions, a standard chromatogram was obtained with symmetry of all peaks ranging from 0.8 to 1.0 (Figure 2).

With regard to optimization of the extraction method for a berry matrix, pure methanol showed higher extractability for all flavonoids investigated than ethanol or acetone as extraction solvents (either pure or mixed with water; Figure S2 in Supplementary Materials). An interesting observation was that pure acetone, which showed a remarkably weak extraction ability in our studies, has previously been reported to exhibit strong extraction ability [15,31]. This discrepancy probably resulted from differences in the sample matrices and the water content of the solvent. Fresh and frozen berries were used as sample matrices in the studies by Garcia-Viguera et al. [31] and Kajdzanoska et al. [15], whereas freeze-dried lingonberry samples, which contain little water were employed in the present study. To investigate the effect of acid on extraction, different amounts of formic acid (1–5%) were added to the extraction solvent (pure methanol and aqueous methanol), which negatively affected the total yield of flavonoids (Figure S3 in Supplementary Materials). As for cyanidin-3-*O*-galactoside, yield was 30% lower on increasing the formic acid concentration to 5%, which might be due to lower stability under the acidic conditions. This finding is consistent with observations by others [15,32,33] who attributed it to instability of flavonoids in extremely acidic environments where hydrolysis, destruction, acetylation, or formylation of polyphenols could occur. Therefore, unacidified methanol was selected as the extraction solvent. In optimization of extraction repetitions, more than 80% of flavonoids in the in-house control sample were found in the first repetition, >10% in the second, less than 5% in the third, and <1% in the fourth (data not shown). Thus, three-repetition extraction, combining supernatants of repetitions 1–3, was selected. 

### 3.2. Quality Control of Quantitative Method

The optimized method provided linearity within the range 2–100 μg/mL, with coefficient of determination (*R*^2^) >0.999 for the 19 compounds (Table 1). The LOD for all compounds ranged between 0.5 and 2.0 μg/mL, which was equivalent to 14.7–59.7 μg/g in freeze-dried berry samples. The LOQ ranged from 1.5 to 6.0 μg/mL, which was equivalent to 43.5–181.2 μg/g in freeze-dried berry samples.

Average recovery on adding an upper and lower level of standard (50% and 100% of the expected content) to the in-house control sample ranged between 84% and 103% for both levels (Table S1 in Supplementary Materials), which is an improvement on the previously reported recovery value for myricetin from lingonberries of 53.2% ± 6% [34].

The intra-day (*n* = 3) and inter-day (*n* = 3) variation (%, CV for peak area) for individual flavonoids in the in-house control sample was 1.7–5.8% and 1.9–7.3%, respectively. 

Standard solutions of all flavonoids investigated (Table 1) maintained stable concentrations of 5 and 50 μg/mL during three months (CV < 10%) at 4 °C, indicating that short-term storage (up to one week) of berry samples in the fridge probably did not significantly affect the outcomes of the analyses.

### 3.3. Flavonoids in Swedish Berries

The content of flavonoids in the berries, quantified using 19 standards, is shown in Table 2. Three compounds (delphinidin-3,5-diglucoside, procyanidin B2, and luteolin-8-*C*-glucoside) were not detected in any of the berry samples. An additional 29 flavonoids in the berry samples were tentatively identified using MS (Table 3) [25,35,36,37] and quantified by UV. 

**Figure 3:**
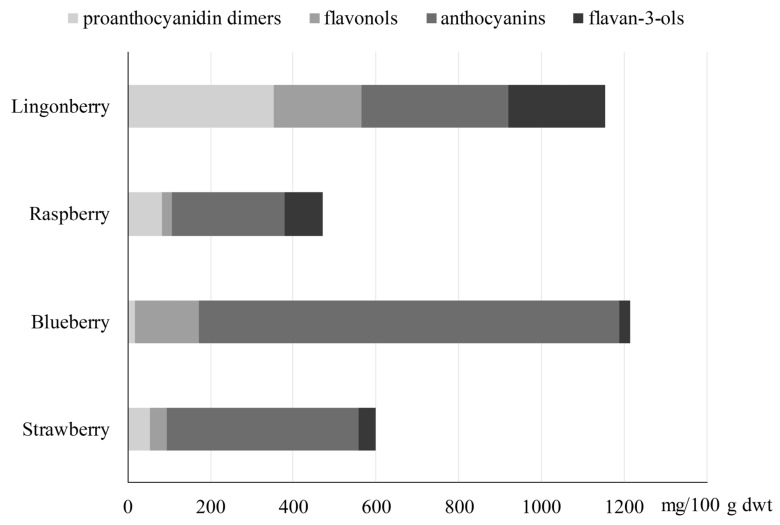
Mean content (mg/100 g dwt) of four subgroups (proanthocyanidins, flavonols, anthocyanins, and flavan-3-ols) of flavonoids in different varieties of lingonberry (*n* = 2), raspberry (*n* = 3), blueberry (*n* = 4), and strawberry (*n* = 7). Analyses were carried out in triplicate.

The average content of proanthocyanidin dimers, flavonols, anthocyanins, and flavan-3-ols in lingonberries, raspberries, blueberries, and strawberries is shown in Figure 3. The tentatively characterized compounds contribute 18%, 29%, 61%, and 67% of the total flavonoid content in strawberries, lingonberries, raspberries, and blueberries, respectively. Blueberries and lingonberries contained the highest amount of flavonoids, and anthocyanins were the dominant flavonoids in all berry samples, ranging from 31% to 84%.

In lingonberries, a total of three anthocyanins, seven flavonols, two flavan-3-ols, and four proanthocyanidins were quantified (Table 2 and Table 3). Cyanidin-3-*O*-galactoside was found to be the most abundant anthocyanin (240–310 mg/100 g dwt) in wild lingonberries, followed by cyanidin-3-*O*-arabinoside (40–80 mg/100 g dwt) and cyanidin-3-*O*-glucoside (10–30 mg/100 g dwt) (Table 2). This is in agreement with Latti et al. [38], who reported content in lingonberries of these three compounds of 267, 57, and 15 mg/100 g dwt, respectively. The content of major flavonols, quercetin-3-*O*-galactoside and quercetin-3-*O*-rhamnoside detected in lingonberry L2 also agreed with the value reported for lingonberry var. Amberland (60 mg/100 g dwt) [39]. Hellstrom and Mattila [40] reported the presence of flavan-3-ols (14 mg/100 g fresh weight (fwt)), proanthocyanidin dimers (29 mg/100 g fwt), and other proanthocyanidins in lingonberries, which was also confirmed by our findings on flavan-3-ols and proanthocyanidin dimers in lingonberry sample L1.

Five anthocyanins, two flavonols, two flavan-3-ols, and one B-type proanthocyanidin were quantified in the raspberry samples (Table 2 and Table 3), and the dominant anthocyanin was tentatively identified as cyanidin-3-*O*-sophoroside confirming finding by others [36,41]. Cyanidin-3-glucosylrutinoside and pelargonidin-3-*O*-rutinoside were only found in Glen Ample in agreement with Sparzak et al. [42], who found differences in polyphenol profile in 11 varieties of red raspberries. Furthermore, the amount of cyanidin-3-*O*-glucoside in var. Glen Ample (Table 2) was in agreement with previous data (34–60 mg/100 g dwt) [43], as well as (−)-epicatechin [44].

Blueberries contained a greater diversity of flavonoids in various amounts, especially anthocyanin compounds, than other berries (Table 2 and Table 3). For the particular var. Duke, our data on the total anthocyanin content agree with the reported value of 1000 mg/100 g dwt [24,45]. 

In strawberries, a total of nine flavonoid compounds, comprising two B-type proanthocyanidins, (+)-catechin, four anthocyanins, and two flavonols, were characterized (Table 2 and Table 3). All varieties had a similar flavonoid profile, but the total content varied greatly from 360–750 mg/100 g dwt. The dominant anthocyanin was confirmed to be pelargonidin-3-*O*-glucoside, but there was an almost four-fold (180–730 mg/100 g dwt) variation between varieties. The dominant flavonol was tentatively identified as quercetin-3-*O*-glucuronide, confirming previous findings [15,28,46]. Wang et al. [46] studied flavonoids in 14 cultivars of strawberry and found the content of anthocyanins to be 450–1000 μg/g fwt, which was confirmed by our results (220–770 mg/100 g dwt, corresponding to 410–1300 μg/g fwt). Other flavonoid compounds (e.g., kaempferol-3-*O*-malonylglucoside) have been reported [15,28] but were not detected in our samples, probably due to differences in the varieties tested.

## 4. Summary

A method enabling the analysis of 45 flavonoid compounds in berry matrices was established. Sixteen flavonoid compounds were quantified with high linearity, precision, and average recovery using external standards. An additional 29 compounds were tentatively identified and quantified using MS. The method was applied for analysis of 16 varieties of Swedish berries.

Both, flavonoid content and pattern were largely dependent on species, but also variety. Blueberries and lingonberries were found to contain 1100 mg/100 g dwt of flavonoids, which is almost twice the content of raspberries and strawberries. Anthocyanins were the dominant flavonoids in all berries. Data should be considered as indicative bearing in mind the limited number of samples and lacking information of postharvest handling. 

## Figures and Tables

**Table 1:** Retention time, wavelength (λ), regression equation, limit of detection (LOD), and limit of quantification (LOQ) during HPLC-UV of flavonoid standards.

No.	Compound	Retention Time (min)	λ ^1^ (nm)	Regression Equation ^2^	LOD ^3^ (μg/mL)	LOQ ^3^ (μg/mL)	Abbre- Viation
1	Procyanidin B1	9.7	280	y = 11.12x − 1.71	0.84	2.56	Pr B1
2	(+)-Catechin	10.1	280	y = 14.10x − 1.72	0.67	2.02	(+)-Ca
3	Delphinidin-3,5-diglucoside	11.6	520	y = 22.35x − 6.10	0.99	2.99	Del-di
4	Procyanidin B2	13.3	280	y = 37.48x − 6.02	0.80	2.41	Pr B2
5	(-)-Epicatechin	15.1	280	y = 11.64x − 2.06	0.85	2.58	(−)-Epi
6	Delphinidin-3-*O*-glucoside	16.7	520	y = 59.52x − 38.58	1.99	6.04	Del-glu
7	Cyanidin-3-*O*-galactoside	17.4	520	y = 64.97x − 12.84	0.96	2.91	Cy-gal
8	Cyanidin-3-*O*-glucoside	19.1	520	y = 57.62x − 12.71	1.09	3.30	Cy-glu
9	Cyanidin-3-*O*-arabinoside	20.5	520	y = 51.51x − 11.54	1.11	3.36	Cy-ara
10	Pelargonidin-3-*O*-glucoside	21.3	520	y = 31.84x − 6.29	0.98	2.96	Pel-glu
11	Petunidin-3-*O*-glucoside	21.8	520	y = 56.17x − 29.61	1.86	5.64	Pet-glu
12	Luteolin-8-*C*-glucoside	24.9	360	y = 53.77x − 4.83	0.49	1.49	Lut-glu
13	Malvidin-3-*O*-glucoside	26.3	520	y = 48.43x − 21.34	1.67	5.06	Mal-glu
14	Procyanidin A2	27.0	280	y = 10.64x − 1.78	0.92	2.80	Pr A2
15	Myricetin-3-*O*-rhamnoside	28.8	360	y = 64.46x − 10.05	0.77	2.34	My-rha
16	Quercetin-3-*O*-galactoside	30.1	360	y = 30.22x − 4.20	0.66	2.01	Qu-gal
17	Quercetin-3-*O*-rutinoside	30.8	360	y = 28.29x − 3.76	0.63	1.90	Qu-rut
18	Quercetin-3-*O*-rhamnoside	35.6	360	y = 39.95x − 6.95	0.89	2.69	Qu-rha
19	Kaempferol-3-*O*-glucoside ^4^	35.6	360	y = 41.27x − 19.89	2.16	6.54	Kae-glu

^1^ Detection wavelength for each compound according to Vagiri et al. [27]. ^2^ Linear range for all compounds was tested 2–100 μg/mL, resulting in *R*^2^ > 0.999. HPLC conditions as described in the section “Quantification”. ^3^ Calculated as: LOD = (3.3 × SD)/b; LOQ = (10 × SD)/b, where SD is residual standard deviation of the linear regression and b is slope of the regression line [29]. ^4^ Kaempferol-3-*O*-glucoside co-eluted with quercetin-3-*O*-rhamnoside, so its calibration curve was built separately.

**Table 2:** Content ^1, 2^ (mg/100 g dwt) of individual flavonoids in selected berry varieties.

	Lingonberry ^3^		Raspberry			Blueberry				Strawberry						
	L1	L2	Kweli	Versalle	Glen Ample	Bluecrop	Duke	Camelia	Legacy	Evie	Favori	Sonata	Faith	Malwina	Salsa	Rumba
**Proanthocyanidins**																
Pr B1	68.1 ± 0.6	111.6 ± 3.8	n.d.	n.d.	n.d.	35 ± 1.3	19.8 ± 0.2	13.9 ± 0.4	n.d.	12.4 ± 1.9	10.9 ± 0.3	15.2 ± 0.1	6 ± 0.1	11.9 ± 0.1	7.9 ± 0.7	12.1 ± 1.1
Pr A2	51.9 ± 0.0	41.8 ± 1.9	n.d.	n.d.	n.d.	n.d.	n.d.	n.d.	n.d.	n.d.	n.d.	n.d.	n.d.	n.d.	n.d.	n.d.
**Flavan-3-ols**																
(+)-Ca	152.7 ± 2.5	243.6 ± 5.9	7.4 ± 0.1	6.1 ± 0.2	4.8 ± 0.2	43.1 ± 1.9	32 ± 0.6	22.6 ± 0.4	10.3 ± 0.6	38 ± 3.1	36.7 ± 1.3	45.8 ± 0.5	30.5 ± 1.3	45.4 ± 1.1	32 ± 1	48.1 ± 1
(-)-Epi	38.6 ± 0.4	34.1 ± 4.4	102.4 ± 4	87.1 ± 2.8	63.9 ± 7.6	n.d.	n.d.	n.d.	n.d.	n.d.	n.d.	n.d.	n.d.	n.d.	n.d.	n.d.
**Flavonols**																
My-rha	n.d.	n.d.	n.d.	n.d.	n.d.	2.5 ± 0.1	2.7 ± 0.2	9.2 ± 0.3	n.d.	n.d.	n.d.	n.d.	n.d.	n.d.	n.d.	n.d.
Qu-gal	35.5 ± 0.0	58.8 ± 2.6	14.3 ± 0.6	19.6 ± 0.3	14.9 ± 0.4	78.6 ± 1.1	67.9 ± 1.6	23.7 ± 1.9	125.8 ± 3.7	n.d.	n.d.	n.d.	n.d.	n.d.	n.d.	n.d.
Qu-rut	n.d.	n.d.	4.1 ± 0.1	7 ± 0.2	9.1 ± 0.3	44.7 ± 2	32.1 ± 1.3	30.4 ± 2.4	19.9 ± 0.6	n.d.	n.d.	n.d.	n.d.	n.d.	n.d.	n.d.
Qu-rha	36.5 ± 0.5	46.1 ± 3	n.d.	n.d.	n.d.	1.3 ± 0.2	1.7 ± 0.2	60.2 ± 3.5	9.4 ± 0.1	n.d.	n.d.	n.d.	n.d.	n.d.	n.d.	n.d.
Kae-glu	n.d.	n.d.	n.d.	n.d.	n.d.	n.d.	n.d.	n.d.	n.d.	9.4 ± 0.5	7.3 ± 0.1	8 ± 0.1	6.1 ± 0.3	5.3 ± 0.1	4.3 ± 0.1	10.1 ± 0.2
**Anthocyanidins**																
Del-glu	n.d.	n.d.	n.d.	n.d.	n.d.	47 ± 2.4	35.1 ± 0.6	33.5 ± 1.4	5.3 ± 0.3	n.d.	n.d.	n.d.	n.d.	n.d.	n.d.	n.d.
Cy-gal	308.4 ± 6.1	238.9 ± 5.1	n.d.	n.d.	n.d.	12.5 ± 0.8	10.5 ± 0.2	7.8 ± 0.5	20.7 ± 1.3	n.d.	n.d.	n.d.	n.d.	n.d.	n.d.	n.d.
Cy-glu	21.1 ± 0.2	17.1 ± 0.7	74.4 ± 0.8	65.2 ± 2.2	55.5 ± 2.8	7.7 ± 0.7	7.2 ± 0.2	3.3 ± 0.1	n.d.	14.3 ± 0.3	11.4 ± 0	6.5 ± 0.5	4.6 ± 0.1	5.3 ± 0.1	9.1 ± 0.5	7.2 ± 0.6
Cy-ara	75.3 ± 0.3	49.8 ± 0.8	n.d.	n.d.	n.d.	67.9 ± 3	47.6 ± 1.1	86.6 ± 4.2	138.4 ± 5.8	n.d.	n.d.	n.d.	n.d.	n.d.	n.d.	n.d.
Pel-glu	n.d.	n.d.	4.5 ± 0.2	2.9 ± 0.3	n.d.	n.d.	n.d.	n.d.	n.d.	562 ± 3.1	487.3 ± 6.9	723.9 ± 15	184.1 ± 7.3	371.4 ± 9.6	278.1 ± 7.1	353.4 ± 8
Pet-glu	n.d.	n.d.	n.d.	n.d.	n.d.	37.7 ± 1.9	29.6 ± 0.9	33.2 ± 1.2	4.9 ± 0.1	n.d.	n.d.	n.d.	n.d.	n.d.	n.d.	n.d.
Mal-glu	n.d.	n.d.	n.d.	n.d.	n.d.	94.3 ± 4.5	83.3 ± 1.8	88.6 ± 3.9	10.5 ± 0.4	n.d.	n.d.	n.d.	n.d.	n.d.	n.d.	n.d.

^1^ Values shown are mean ± SD (*n* = 3). Abbreviations and full names of compounds can be found in Table 1. ^2^ Delphinidin-3,5-diglucoside, procyanidin B2, and luteolin-8-*C*-glucoside were not detected in any of the berry samples. ^3^ Wild lingonberry samples bought from two producers were named L1 and L2 due to a lack of information on variety. Recovery for flavonoids detected in the control sample lingonberry L1 was between 84% and 103% (Table S1 in Supplementary Materials). The moisture content of the berry samples ranged between 80.1% and 87.5% (see Table S2 in Supplementary Materials).

**Table 3:** Content (mg/100 g dwt) of tentatively identified flavonoids in all berries.

Compound	RT ^1^ (min)	[M+H] ^+^ (*m/z* ^2^)	Berry Varieties (mg/100 g dwt)							
			Lingonberry							
			L1				L2			
**Proanthocyanidins**										
B-type procyanidin ^3^	8.8	579	46.7 ± 3				66 ± 6.4			
A-type procyanidin ^4^	22.2	577	130 ± 1.5				188.6 ± 5.5			
**Flavonols ^5^**										
Quercetin-3-*O*-xyloside	32.7	435	7.1 ± 0.3				10.8 ± 0.5			
Quercetin-3-*O*-arabinoside	33.7	435	6.3 ± 0.1				9 ± 0.4			
Quercetin-3-*O*-arabino-furanoside	34.3	435	42.5 ± 1.9				56 ± 2.2			
Quercetin-3-*O*-(4’’-HMG)-rhamnoside ^6^	43.6	593	41.8 ± 1.1				64.3 ± 2.9			
Kaempferol-(HMG)-rhamnoside	49.6	577	4.6 ± 0.1				3.4 ± 0.2			
			Blueberry							
			Bluecrop	Duke			Camelia			Legacy
**Anthocyanidins ^7^**										
Delphinidin-3-*O*-galactoside	15.4	465	88.8 ± 3.7	61.3 ± 1.4			110.9 ± 5.4			164 ± 6.8
Delphinidin-3-*O*-arabinoside	18.4	435	87.1 ± 5.2	67.5 ± 1.3			82.3 ± 3.5			80.8 ± 2.9
Petunidin-3-*O-*galactoside	20.5	479	60.8 ± 2.7	42.6 ± 1			77.5 ± 3.7			123.8 ± 5.1
Peonidin-3-*O*-galactoside	23.2	463	4.4 ± 0.8	3.8 ± 0.2			2.1 ± 0.2			n.d.
Petunidin-3-*O*-arabinoside	23.6	449	41.5 ± 2.2	28.9 ± 0.8			45.8 ± 2.2			47.4 ± 1.3
Peonidin-3-*O-*glucoside	24.2	463	5.4 ± 0.7	6.1 ± 0.1			4.2 ± 0.3			0.4 ± 0.4
Malvidin-3-*O*-galactoside	25.0	493	132.9 ± 6.2	87.9 ± 2.5			187.5 ± 8.7			289.8 ± 14
Peonidin-3-*O-*arabinoside	25.7	433	14.2 ± 0.9	8.5 ± 0			3.7 ± 0.1			0.4 ± 0.4
Malvidin-3-*O*-arabinoside	28.2	463	141.7 ± 6.1	95.4 ± 2.3			121.2 ± 6.7			136.5 ± 5.3
Delphinidin-3-acetyl-glucoside	29.9	507	16.5 ± 0.6	8 ± 0			2.4 ± 0			n.d.
Petunidin-3-acetyl-glucoside	35.0	521	15.7 ± 0.6	8.5 ± 0.6			n.d.			n.d.
Cyanidin-3-malonyl-glucoside	35.2	535	27.7 ± 0.9	10.3 ± 0.4			n.d.			n.d.
Malvidin-3-acetyl-glucoside	39.2	491	43.6 ± 1.4	19.9 ± 0.4			3.3 ± 0.3			n.d.
**Flavonols ^5^**										
Myricetin-3-*O*-arabinoside	32.3	465	12.1 ± 0.6	10.6 ± 0.3			n.d.			8.5 ± 0.6
Quercetin-3-*O-*arabinoside	33.6	435	20.2 ± 0.6	19.5 ± 0.7			7.3 ± 0.7			28.9 ± 1.1
			Raspberry							
			Kweli		Versalle				Glen Ample	
**Proanthocyanidins**										
B-type procyanidin^3^	11.5	579	80 ± 1.6		123.8 ± 3.6				43.1 ± 4.1	
**Anthocyanidins ^7^**										
Cyanidin-3-*O*-sophoroside	17.2	611	192.4 ± 3.2		210.9 ± 6.5				81.6 ± 4.1	
Cyanidin-3-glucosyl-rutinoside	19.3	757	n.d.		n.d.				82.9 ± 8.9	
Pelargonidin-3-*O*-rutinoside	21.6	579	n.d.		n.d.				51.2 ± 2.5	
			Strawberry							
			Evie	Favori	Sonata	Faith		Malwina	Salsa	Rumba
**Proanthocyanidins**										
B-type procyanidin ^3^	8.8	579	31.5 ± 2.2	22.7 ± 2.3	34.8 ± 4.6	57.7 ± 1.8		33.1 ± 1.3	65.8 ± 1.6	40.2 ± 0.3
**Anthocyanidins ^7^**										
Cyanidin-hexose-deoxyhexoside	24.1	595	6.8 ± 0.2	7.9 ± 1.0	9.8 ± 0.2	5.7 ± 0.1		7.6 ± 0.2	11.8 ± 0.4	8.8 ± 0.3
Pelargonidin-3-*O*-malonylglucoside	30.6	519	22.4 ± 0.2	20.3 ± 0.2	30.1 ± 0.9	23.8 ± 0.9		12.6 ± 0.4	20.3 ± 0.6	38.2 ± 0.8
**Flavonols ^5^**										
Quercetin-3-*O*-glucuronide	30.7	479	50.7 ± 1.6	28.5 ± 0.3	21.9 ± 1.4	40.5 ± 1.1		22.8 ± 0.5	39.1 ± 1.7	47.8 ± 1.5

^1^ RT: retention time. ^2^ *m/z*: mass-to-charge ratio. ^3^ Quantified using procyanidin B1 standard. ^4^ Quantified using procyanidin A2 standard. ^5^ Quantified using quercetin-3-*O*-galactoside standard. ^6^ HMG: 3-hydroxy-3-methylglutaroyl. ^7^ Quantified using cyanidin-3-*O*-glucoside standard. The moisture content of the berry samples ranged between 80.1% and 87.5% (see Table S2 in Supplementary Materials).

## Supplementary Materials

The following are available online at https://www.mdpi.com/2304-8158/9/3/358/s1, Figure S1: Effect of formic acid concentration (1–12%) on separation of flavonoids (5 μg/mL) at 360 nm, Figure S2: Effect of solvent on extraction yield, Figure S3: Effect of formic acid concentration in solvent on extraction yield, Table S1: Recovery rate of different flavonoids in lyophilized lingonberries, Table S2: Moisture content in the 16 berry varieties analyzed.
