# Supplementary material for: Characterization of Flavonoid Compounds in Common Swedish Berry Species
Source: Foods. 2020 Mar 19;9(3):358. doi: 10.3390/foods9030358 (PMC7143522; doi:10.3390/foods9030358)
Supplement: Supplementary file 1 [file foods-09-00358-s001.pdf]

Supplementary Materials

Table S1. Recovery rate of different flavonoids in lyophilized lingonberries

Compound	C_{sample} [1] (mg/100g)	C_{added} (mg/100g)	C_{found} [1] (mg/100g)	Recovery [2] (%)	Average (%)
Pr B1	63.2±1.1	64.9	126.9±0.7	98.2±2.8	97.1
		32.6	94.5±1.2	96.0±7.1	
(+)-Ca	156.2±5.3	152.9	301.1±1.9	94.8±4.7	89.6
		77.7	221.7±0.6	84.3±7.6	
(-)-Epi	35.4±1.7	33.2	68.3±0.5	99.1±6.6	93.2
		16.5	49.8±0.4	87.3±12.7	
Pr A2	55.7±1.8	52.8	105.9±0.2	95.1±3.8	92.7
		26.4	79.5±1.2	90.2±11.4	
Qu-gal	35.9±1.5	38.3	75.2±2.2	102.6±9.7	97.5
		19.6	54.0±0.6	92.3±10.7	
Qu-rha	37.0±1.3	40	77.1±0.5	100.3±4.5	96.3
		20.4	55.8±0.8	92.2±10.3	
Cy-gal	315.8±5.5	282.3	597.9±5.8	99.9±4.0	95.6
		139.1	442.8±6.9	91.3±7.5	
Cy-glu	22.4±0.4	26.6	48.4±0.2	97.7±2.3	94
		13.3	34.4±0.0	90.2±3.0	
Cy-ara	78.1±2.2	73.7	149.2±2.7	96.5±6.6	93.9
		36.3	111.2±1.5	91.2±10.2	

[1] Values shown are mean ± SD (n = 2). *C* refers to concentration. Abbreviations and full names of compounds are presented in Table 1; [2] Calculated as: Recovery (%) = $(C_{found}-C_{sample})\times100/C_{added}$, see section 2.5 in the main text.

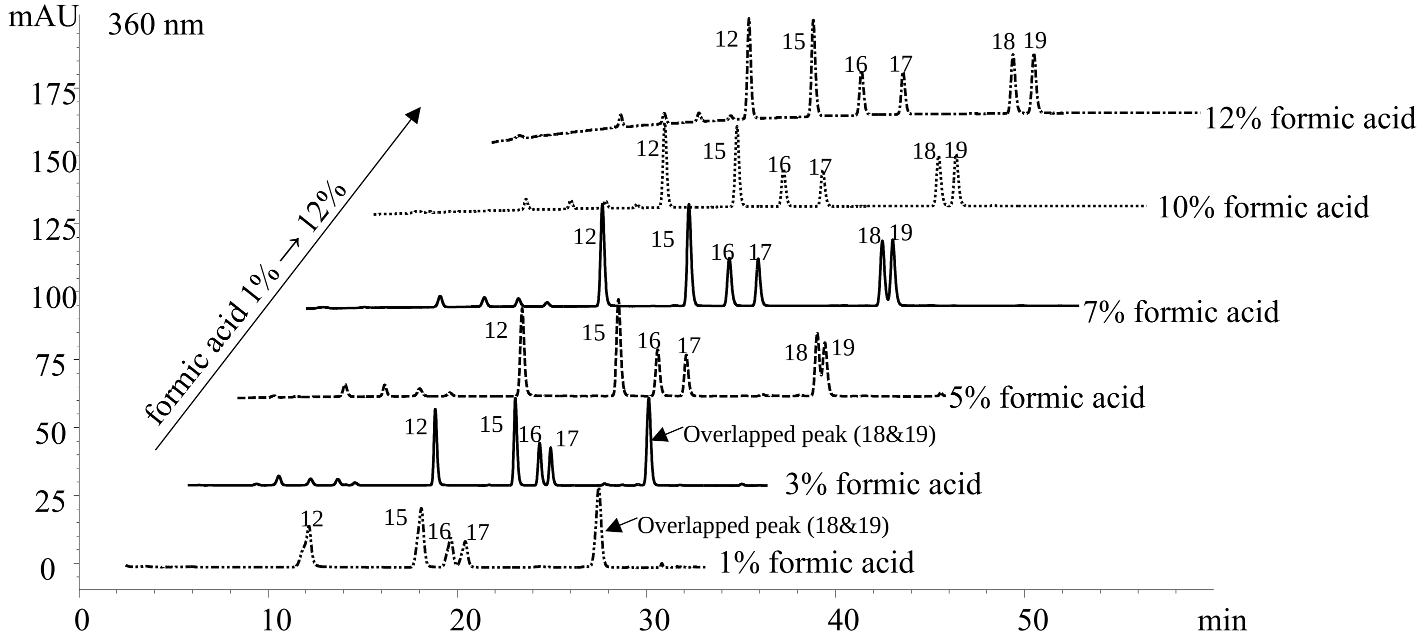

Figure S1. Effect of formic acid concentration (1–12%) on separation of flavonoids (5 μg/mL) at 360 nm. HPLC conditions and peak number as described in section 2.4 and Table 1, respectively. The last peak in the lower panel is for compounds 18 and 19, which were separated in the chromatograms displayed in the upper panels.

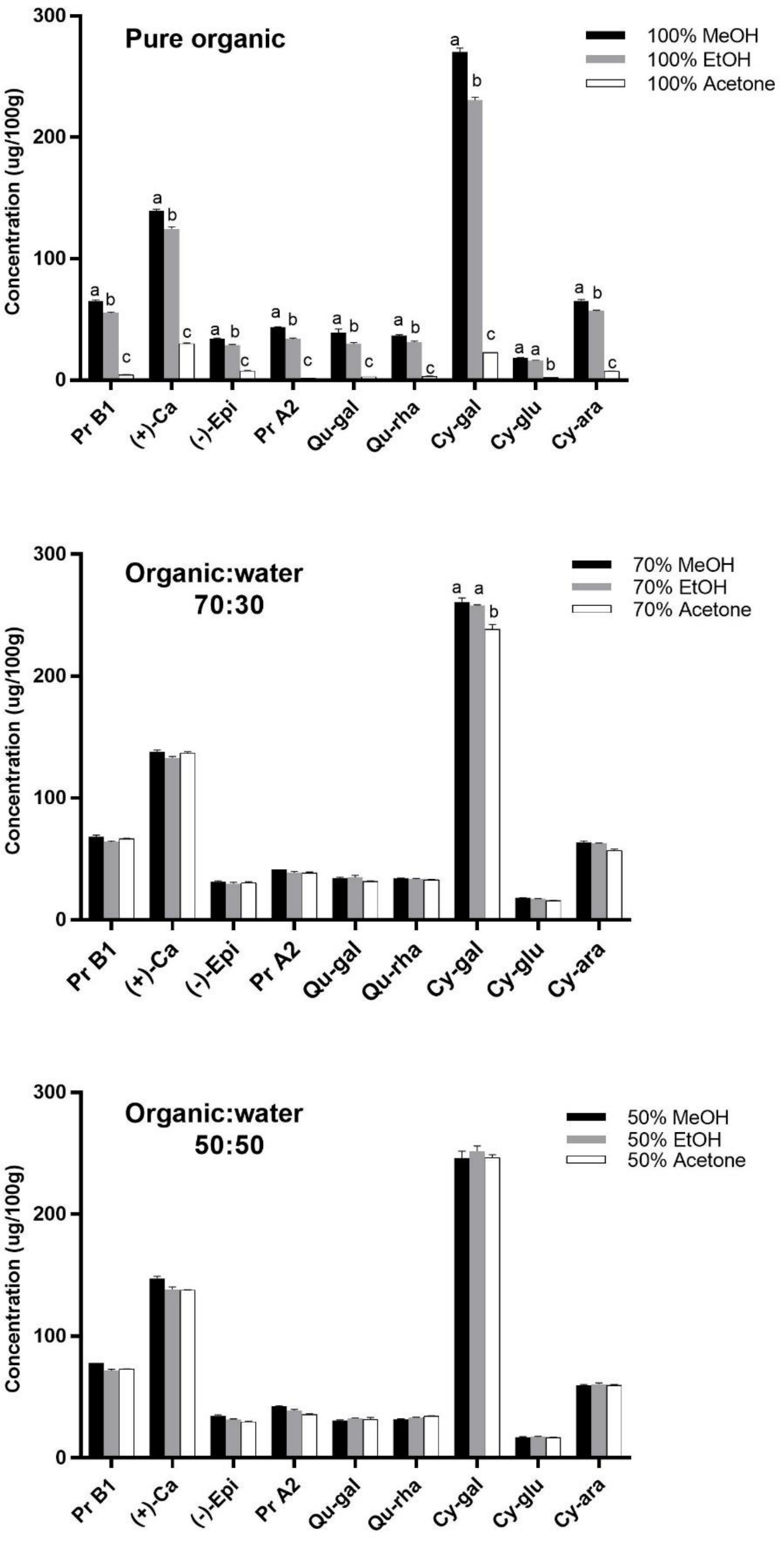

Figure S2. Effect of solvent on extraction yield. In each panel, different letters above columns representing the same compound indicate significant difference ($p < 0.05$). Columns represent means of duplicate analysis. Abbreviations refer to Table 1.

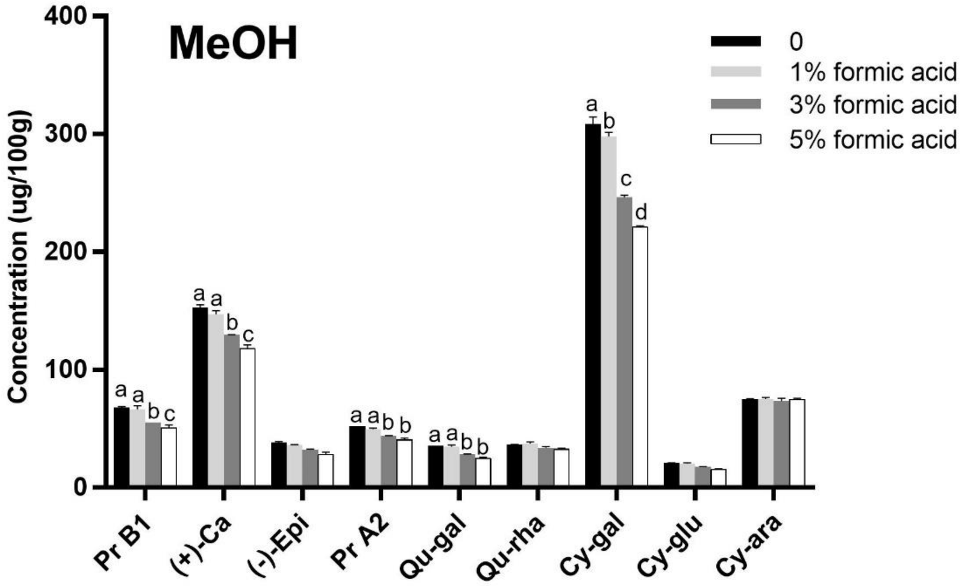

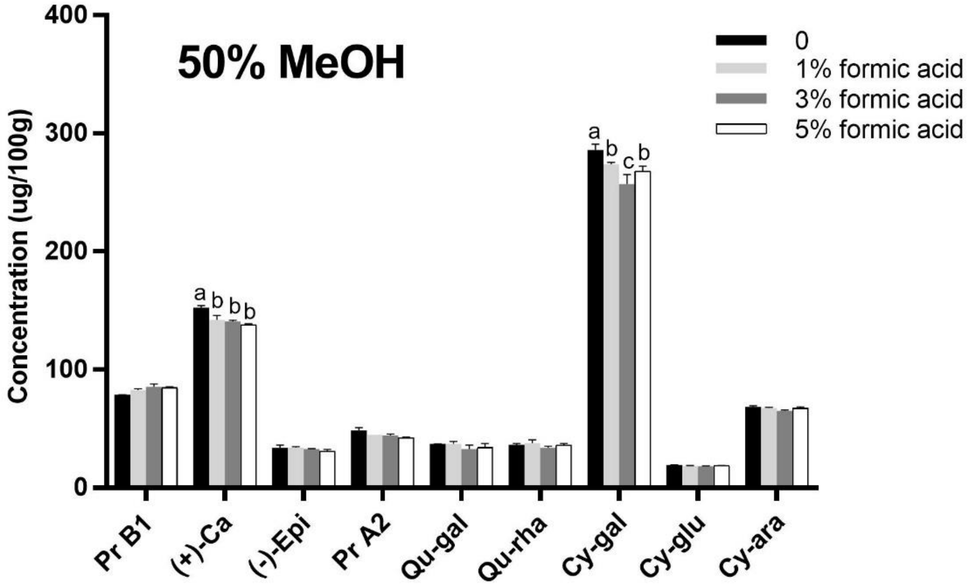

Figure S3. Effect of formic acid concentration in solvent on extraction yield. In each panel, different letters above columns representing the same compound indicate significant difference ($p < 0.05$). Columns represent means of duplicate analysis. Abbreviations refer to Table 1.

Table S2. Moisture content [1] in the 16 berry varieties analyzed.

Berry Species	Variety/Sample Code	Moisture Content (%)
Lingonberry	L1	80.3
	L2	81.1
Raspberry	Kweli	82.6
	Versalle	82.7
	Glenampel	84.9
Blueberry	Legacy	81.1
	Bluecrop	81.7
	Duke	84.0
	Camelia	80.1
Strawberry	Evie	84.9
	Favori	80.4
	Sonata	83.2
	Faith	83.1
	Malwina	81.9
	Salsa	87.3
	Rumba	87.5

[1] Moisture content (MC) calculated as: MC = (FW−DW)/FW, where FW is fresh weight, and DW is dry weight after freeze drying.
